# Evaluation of *Bacillus subtilis* PB6 on feedlot phase growth performance, efficiency of dietary net energy utilization, and fecal and subiliac lymph node *Salmonella* prevalence in spring placement yearling beef steers fed in southeastern South Dakota^1,2,3^

**DOI:** 10.1093/tas/txab002

**Published:** 2021-01-09

**Authors:** Zachary K Smith, Paul Rand Broadway, Keith R Underwood, Warren C Rusche, Julie A Walker, Nicole C Burdick Sanchez, Jeffrey A Carroll, Doug Lafleur, Jerilyn E Hergenreder

**Affiliations:** 1 Department of Animal Science, South Dakota State University, Brookings, SD, USA; 2 USDA-ARS Livestock Issues Research Unit, Lubbock, TX, USA; 3 Kemin Industries, Des Moines, IA, USA

**Keywords:** *Bacillus subtilis*, beef, feedlot, probiotic, *Salmonella*

## Abstract

Yearling crossbred beef steers [*N* = 238; initial shrunk body weight (BW) = 402 ± 31.2 kg] were used to investigate the influence of a *Bacillus subtilis* probiotic on animal growth performance, efficiency of dietary net energy (NE) utilization, carcass characteristics, and fecal and subiliac lymph node *Salmonella* prevalence during a 140-d finishing period at the Southeast Research Farm in Beresford, SD. Steers were allotted to 1 of 24 pens (*N* = 9–10 steers/pen) and assigned to 1 of 2 dietary treatments (12 pens/treatment): no probiotic (CON) or 0.5 g/steer/d of a *B. subtilis* PB6 probiotic (CLOSTAT500, Kemin Industries, Des Moines, IA; CLO). Bunks were managed according to a slick bunk management approach. Fecal samples were collected on study days 1, 28, 56, 112, and 140 from a subsample of steers from each pen (*N* = 5 steers/pen) via rectal palpation and composited by pen for the determination of *Salmonella* prevalence using selective enrichment and culture media. Upon harvest, subiliac lymph nodes were obtained from an equal number of steers from each treatment (collected from every other steer) following evisceration and hide removal. Data were analyzed as a randomized complete block design and pen served as the experimental unit; an α of 0.05 determined significance. Live-basis final BW and average daily gain tended (*P* ≤ 0.06) to be reduced for CLO. No differences were detected (*P* ≥ 0.11) between treatments for dry matter intake or gain efficiency. Treatment neither altered the efficiency of dietary NE utilization nor calculated dietary NE content based upon observed performance (*P* ≥ 0.46). No differences were detected between treatments for any carcass traits (*P* ≥ 0.15). No *Salmonella* was recovered in any fecal samples collected on study days 1, 28, or 56. On day 112, steers from CLO had a numerically lower (*P* = 0.17; 25.0 vs. 8.3%) incidence of fecal *Salmonella* compared to CON. On study day 140, fecal *Salmonella* incidence did not differ between treatments (*P* = 0.34; 0.0 vs. 8.3%) for CON and CLO, respectively. Upon harvest, no *Salmonella* was recovered in any subiliac lymph nodes. These data indicate that *B. subtilis* PB6 did not influence feedlot phase growth performance or fecal *Salmonella* prevalence. Additionally, *Salmonella* was not observed in the subiliac lymph nodes of any steers upon harvest.

## INTRODUCTION

Food safety is an issue of concern for producers, consumers, and processors of livestock products. Foodborne pathogens, such as *Salmonella*, can result in human disease. Recently, there have been efforts to include specific *Salmonella* serotypes as adulterants in raw beef products (Gremillion, 2018). Furthermore, as of summer 2020, U.S. Department of Agriculture–Food Safety and Inspection Service (USDA–FSIS) has received a citizen petition asking to declare 31 *Salmonella* serotypes as adulterants of meat and poultry products (FSIS Salmonella Petition 1.19.20). *Salmonella* can infect the gastrointestinal tract of beef animals at a variety of time points in the life of the beef animal (Gragg et al., 2013; Broadway et al., 2020). In the United States, there are regional differences in *Salmonella* prevalence in fed-cattle populations (Green et al., 2010; Gragg et al., 2013). Steers fed and harvested in the Northern Plains region of the United States (e.g., South Dakota) have been shown to have little to no *Salmonella-*positive lymph nodes upon harvest (Gragg et al., 2013). *Salmonella* can proliferate in the gastrointestinal tract and subsequently takes residence in subiliac lymph nodes where it can then contaminate beef trim (Gragg et al., 2013; Gremillion, 2018). Many cattle may harbor and shed *Salmonella* but remain asymptomatic; however, there is still the risk of reduced feed intake and growth performance in these cattle infected with *Salmonella*. Currently, many antimicrobial alternatives are being investigated to determine their preharvest efficacy to reduce foodborne pathogens (Broadway et al., 2014). One of the primary goals of the feedlot industry is to increase animal growth performance and gain efficiency during all stages of the feedlot production phase. Production enhancement technologies (e.g., steroidal implants with anabolic activity and beta-adrenergic agonist) are routinely employed in North American feedlots to increase production efficiencies (Johnson et al., 2013; Smith and Johnson, 2020). Additionally, feed grade and injectable antimicrobials are used in North American beef production to prevent and treat illness in cattle. The safety of these production enhancement technologies, feed grade, and injectable antimicrobials has been confirmed through many thorough evaluations; however, there is still widespread concern surrounding the safety of these products amongst consumers (Sánchez-Mendoza et al., 2014). Thus, there has been considerable attention focused on nonpharmaceutical alternatives to the use of these compounds in food production (Sánchez-Mendoza et al., 2014). *Bacillus subtilis* PB6 (CLOSTAT500, Kemin Industries, Des Moines, IA) is a patented spore-forming bacterium that has been shown to impact clostridia and *Salmonella* in livestock species (Broadway et al., 2020; Smock et al., 2020b). Reducing subclinical illness in livestock that is associated with clostridia and *Salmonella* challenges can, in turn, improve immunological responses to more severe diseases associated with the respiratory tract in cattle. This ultimately could reduce the need for therapeutic and subtherapeutic administration of antimicrobials during the feedlot production phase and, in turn, enhance growth performance and growth efficiency (Broadway et al., 2020). The objective of this research was to determine the influence of *B. subtilis* PB6 administration in yearling feedlot steers on growth performance, efficiency of dietary net energy (NE) utilization, carcass trait responses, and *Salmonella* prevalence.

## MATERIALS AND METHODS

Animal care and handling procedures used in this study were approved by the South Dakota State University Animal Care and Use Committee (approval number: 2003-019E). The study was conducted at the Southeast Research Farm (SERF) Feedlot located near Beresford, SD (43.0805°N, 96.7737°W).

### Dietary Treatments

This study used 12 replicate pens of 9–10 steers/pen assigned to one of two dietary treatments. Dietary treatments included:

1) No probiotic (CON).
2) Fed 0.5 g/steer/d of a *B. subtilis* PB6 probiotic (CLOSTAT 500, Kemin Industries, Des Moines, IA; CLO).

### Animals, Initial Processing, and Study Initiation

A total of 238 crossbred beef steers (initial BW 402 ± 31.2 kg) were used in this study. Steers were sourced from a grow yard in northwest Iowa and transported 160 km to the SERF on March 17, 2020. All steers were processed on March 20, 2020. At the time of initial processing, individual body weight (BW) was collected, and a unique identification tag was applied to each steer. Steers were also vaccinated against respiratory pathogens: infectious bovine rhinotracheitis, bovine viral diarrhea types 1 and 2, parainfluenza-3 virus, and bovine respiratory syncytial virus (Bovi-Shield Gold 5, Zoetis, Parsippany, NJ) and clostridial species (ULTRABAC 7/Somubac, Zoetis), administered pour-on moxidectin (Cydectin, Bayer Healthcare LLC, Pittsburgh, PA) and administered a steroidal implant (200 mg trenbolone acetate and 28 mg estradiol benzoate; SYNOVEX PLUS, Zoetis). On study day 28, all steers were revaccinated for clostridial species (ULTRABAC 7/Somubac, Zoetis). The study was initiated on March 23, 2020 (6 d following arrival to the SERF). Steers were housed in open-lot, soil-surfaced pens, with 6.1 m of bunk space, a 6.0 m concrete bunk apron, and 60.4 or 54.4 m^2^ of pen space per steer (9 or 10 steers/pen).

### Weather Measurement and Temperature–Humidity Index Estimation

Climatic variables [ambient temperature (*T*_*a*_), relative humidity (RH), and wind speed] were obtained every 5 min from a weather station (Mesonet at SDState) located at the SERF throughout the experimental period. The temperature–humidity index (THI) was calculated using the following formula: THI = 0.81 × *T*_*a*_ + [RH × (*T*_*a*_ − 14.40)] + 46.40 (Hahn, 1999).

### Diet and Intake Management

Steers were fed once daily in the morning. Bunks were managed to be slick at 0700 h most mornings. Steers were stepped up to their final diet over a 14-d period with two step-up diets fed. Feed intake and diet formulations were summarized at weekly intervals. Steers were fed common diets only differing in regards to the addition of the *B. subtilis* PB6 probiotic (Table 1). Individual ingredient samples (except for the dietary treatment pellet and liquid supplement) were collected weekly and dry matter (DM) calculated after drying in a forced-air oven at 60 °C until no further weight change to determine the DM intake (DMI). Proximate analysis of each ingredient (except for pelleted treatment supplement and liquid supplement) was conducted weekly according to: DM [method no. 935.29 (AOAC, 2012)], N [method no. 968.06 (AOAC, 2016); Rapid Max N Exceed; Elementar; Mt. Laurel, NJ], and ash [method no. 942.05 (AOAC, 2012)]. Modified distillers grains samples were analyzed for ether extract content using an Ankom Fat Extractor (XT10; Ankom Technology, Macedon, NY) and tabular values for the remainder of the ingredients were used (NASEM, 2016). Percentages of acid detergent fiber (ADF) and neutral detergent fiber (NDF) were assumed to be 3% and 9% for corn, respectively. Analysis of ADF and NDF composition for all other ingredients was conducted as described by Goering and VanSoest, 1970.

**Table 1.** Actual diet formulation fed and nutrient composition from weekly ingredient analyses^*a*^

	Days fed						
Item	1–7	8–14	15–21	22–56	57–74	75–116	117–140
Dry-rolled corn, %	39.00	48.75	64.23	64.94	64.20	68.87	69.33
Modified distillers grains plus solubles, %	20.01	20.82	14.19	16.57	18.26	17.42	17.27
Alfalfa-grass hay blend, %	29.49	17.54	5.03	–	–	–	–
Millet hay, %	–	–	–	2.92	3.05	7.90	–
Corn silage, %	4.74	5.69	10.27	9.30	8.56	–	–
Grass hay, %	–	–	–	–	–	–	7.58
Liquid supplement^*b*^, %	3.90	4.11	4.03	4.02	3.96	3.88	3.88
Pelleted treatment supplement^*c*^, %	2.86	3.09	2.25	2.25	1.97	1.93	1.94
Diet DM, %	68.46	66.28	65.48	64.62	65.62	76.51	76.46
Crude protein, %	13.73	13.33	11.29	12.17	12.11	12.83	12.51
NDF, %	34.92	28.83	19.95	18.79	18.15	18.77	17.96
ADF, %	19.79	15.62	10.24	9.88	9.22	9.53	9.46
Ash, %	7.18	6.43	4.90	5.27	5.55	5.58	5.36
Ether extract, %	3.31	3.51	3.60	4.47	4.79	4.42	4.28
NE for maintenance, Mcal/kg	1.87	1.97	2.05	2.07	2.08	2.07	2.09
NE for gain, Mcal/kg	1.19	1.29	1.39	1.40	1.41	1.40	1.41

^*a*^All values except diet DM on a DM basis.

^*b*^The liquid supplement contained (all values except for DM on a DM basis): 69.05% DM, 25.76% crude protein, 22.42% nonprotein nitrogen, 0.88 Mcal/kg of NE for maintenance, 0.64 Mcal/kg of NE for gain, 59.60% ash, 17.00% calcium, 0.24% phosphorus, 1.26% potassium, 0.07% magnesium, 7.24% NaCl, 0.27% sulfur, 2.66 ppm cobalt, 466.63 ppm inorganic copper, 40.14 ppm iodine, 10.49 mg/kg EDDI, 57.14 ppm iron, 832.71 ppm manganese, 4.05 ppm selenium, 2,498.14 ppm inorganic zinc, 11,078.69 ppb chromium propionate, 478.28 ppm organic copper complex, 1,560.86 organic zinc complex, 31,038.38 IU/kg vitamin A, 170.79 IU/kg vitamin E, and 805.95 g/Mg monensin sodium.

^*c*^Pelleted treatment supplement consisted of exclusively soybean hulls for control (CON) steers and soybean hulls plus CLOSTAT 500 (CLO; Kemin Industries, Des Moines, IA) at 2,080 g/Mg sufficient to provide 0.5 g/steer/d for CLO steers.

Weekly DM and assayed nutrient composition values were used to tabulate actual DM ingredient inclusions and assayed nutrient composition of the diets fed along with tabular ingredient energy values presented in Table 1 according to NASEM, 2016.

### Cattle Management and Growth Performance Parameters

Steer BW was recorded at the time of study initiation and on days 28, 56, 84, 112, and 140 for the calculation of average daily gain (ADG) and feed conversion efficiency [gain:feed; (G:F)]. Body weights were measured before the morning feeding and a 4% pencil shrink was applied to initial BW and final BW (BW from day 140) for the calculation of cumulative steer growth performance. Carcass-adjusted final BW was calculated from hot carcass weight (HCW)/0.625 for the calculation of carcass-adjusted growth performance.

Carcass-adjusted growth performance was used to calculate performance-based dietary NE to determine the efficiency of dietary NE utilization. The performance-based dietary NE was calculated from daily energy gain (EG; Mcal/d): EG = ADG^1.097^ × 0.0557W^0.75^, where W is the mean equivalent shrunk BW [kilograms (NRC, 1996)] from median feeding shrunk BW and final BW at 28% estimated empty body fatness (AFBW) calculated as: [median feeding shrunk BW × (478/AFBW), kg (NRC, 1996)]. Maintenance energy (EM) was calculated by the equation: EM = 0.077 × BW^0.75^. Dry matter intake is related to energy requirements and dietary NEm (Mcal/kg) according to the following equation: DMI = EG/(0.877NEm − 0.41) and can be resolved for the estimation of dietary NEm by means of the quadratic formula $x=\frac{-b\pm \sqrt{b^{2}-4ac}}{2c}$, where *a* = −0.41EM, *b* = 0.877EM + 0.41DMI + EG, and *c* = −0.877DMI (Zinn and Shen, 1998). Dietary NEg was derived from NEm using the following equation: NEg = 0.877NEm − 0.41 (Zinn, 1987).

### Management of Pulls and Removals

All steers that were pulled from their home pen for health evaluation were then monitored in individual hospital pens prior to being returned to their home pens. When a steer was moved to a hospital pen, the appropriate amount of feed from the home pen was removed and transferred to the hospital pen. If the steer in the hospital returned to their home pen, this feed remained credited to the home pen. If the steer did not return to their home pen, all feed that was delivered to the hospital pen was deducted from the feed intake record for that particular pen back to the date the steer was hospitalized. Four steers died during the course of the experiment for reasons determined to be health anomalies not related to dietary treatment. Two steers from CON died of heart failure, and two steers from the CLO died due to pneumonia associated with bovine respiratory disease complex.

### Study Termination and Carcass Data Collection

The study was terminated on August 10, 2020, when steers were visually appraised to have 1.27 cm of rib fat (RF). All steers were shipped (99 km) the same day as study termination and harvested the following day at Tyson Fresh Meats in Dakota City, NE. Steers were maintained by treatment during shipping and lairage and subsequently harvested as two individual lots in order to ensure that an adequate number of subiliac lymph nodes were collected from each treatment. Individual steer identity was tracked through the harvest facility by trained personnel from South Dakota State University. Hot carcass weight and liver abscess scores were recorded during the harvest procedure. Liver scores were classified according to the Elanco Liver Scoring System: normal (no abscesses), A− (one or two small abscesses or abscess scars), A (two to four well-organized abscesses less than 2.54 cm diameter), or A+ (one or more large active abscesses greater than 2.54 cm diameter with inflammation of surrounding tissue). Video image data were obtained from the abattoir for ribeye area (REA), RF, kidney-pelvic-heart fat (KPH), and USDA marbling scores. Dressing percentage was calculated as: (HCW/final BW shrunk 4%) × 100. Estimated empty body fat (EBF) percentage and AFBW were calculated from observed carcass traits (Guiroy et al., 2002). Yield grade (YG) was calculated according to the USDA regression equation (USDA, 1997). Estimated proportion of closely trimmed boneless retail cuts from carcass round, loin, rib, and chuck (Retail Yield; RY) was also calculated from carcass traits (Murphey et al., 1960).

### *Salmonella* Prevalence Determination

Fecal grab samples were aseptically collected via rectal palpation during the weighing procedure, from the same steers throughout the course of the study, at study initiation and on days 28, 56, 112, and 140 (6, 34, 62, 118, and 146 d following arrival to the SERF) according to Broadway et al. (2020). Briefly, samples were obtained from the five steers closest to the initial pen mean average from each of the 24 pens (12 pens/treatment). Samples were aseptically transferred to sealable bags and shipped overnight to USDA–ARS in Lubbock, TX, in shipping coolers containing enough ice packs to keep the temperature of the samples between 0 and 4 °C. Upon arrival, samples were weighed, and an equal portion of feces from each steer was pooled by pen and homogenized. Twenty-five grams of the pooled sample was added to 225 mL phosphate buffered saline (PBS) and homogenized for 2 min in a stomacher. From the strainer bag, 1 mL of the homogenate was removed and placed into a 1.5-mL microcentrifuge tube and subjected to serial dilution in PBS. Then, 100 uL of selected dilutions were plated via stick-spreading on Brilliant Green Agar and incubated overnight at 37 °C. After incubation, phenotypical colonies were counted to determine *Salmonella* concentrations defined as colony-forming units per gram based on the dilution factor. Phenotypic colonies from each plate were pulled and subjected to a *Salmonella* latex agglutination test for *Salmonella* confirmation. In conjunction with quantification, 1 mL of homogenate was placed in a 1:10 dilution of Tetrathionate Broth with iodine and Rappaport Vassiladus enrichment broth and placed in 37 and 42 °C incubators, respectively, overnight. From the enriched cultures, a 10-uL loop was utilized to streak the enriched media onto brilliant Green and XLT4 agars. Similarly, latex agglutination was performed on phenotypic colonies. Subiliac lymph nodes were collected from every other carcass during the harvest procedure. Samples were denuded and subjected to similar procedures as outlined above for the determination of *Salmonella*.

### Statistical Analysis

Growth performance data were analyzed as a randomized complete block design using the MIXED procedure of SAS 9.4 (SAS Inst. Inc., Cary, NC) with pen as the experimental unit. The model included the fixed effect of dietary treatment; block (location) was included as a random variable. Least squares means were generated using the LSMEANS statement of SAS. Treatment means were compared using the *F*-test statistic. An α of 0.05 or less determined significance and tendencies were declared from 0.051 to 0.10.

## RESULTS AND DISCUSSION

### Weather Measurements

Ambient weather conditions during the course of the study are presented in Table 2. Average THI during the course of the 140-d study was 61.6. The THI was above 75 for 21 d of the 140-d study. The average total precipitation at the SERF for the past 67 y from March to August is 455.7 mm. The precipitation during the course of this experiment was below historical records. Two separate heat events occurred during period 4 (mean period THI = 72.5) of the present study (days 85–112) in which the average THI was greater than 75 for 10 d of the 28-d period.

**Table 2.** *T*_*a*_, mean RH, and THI throughout the course of the experiment

Period^*a*^	Mean *T*_*a*_ (°C)	Mean RH (%)	Mean THI^*b*^	Days with THI >75	Wind speed, KPH	Total precipitation, mm
Pretrial (6 d)	0.5	83.9	35.4	0	15.8	20.8
1	4.6	73.4	42.7	0	14.4	56.9
2	11.8	64.5	54.1	0	12.5	33.3
3	20.9	67.6	67.2	4	15.0	45.0
4	23.6	77.4	72.5	10	10.8	89.2
5	22.5	80.9	71.2	7	9.1	52.8
Average^*c*^	16.7	72.7	61.6	21	12.4	279.9

^*a*^Each period represents 28 d.

^*b*^THI = 0.81 × *T*_*a*_ + [RH × (*T*_*a*_ − 14.40)] + 46.40.

^*c*^Average of the 140-d study, except for days with THI >75 and precipitation, which is total days with THI >75 and total precipitation during the course of the 140-d study.

### Animal Growth Performance

Animal growth performance responses for the 140-d study are located in Table 3. There was no difference detected for initial on test BW (*P* = 0.37; 402 vs. 401 ± 1.29 kg) for CON and CLO steers, respectively. Final BW and ADG (live basis) from the 140-d experiment tended to be decreased (*P* ≤ 0.06) for CLO steers compared to CON steers. Dietary treatment had no influence on live-basis G:F. Dry matter intake was not different (*P* = 0.63) between treatments. Carcass-adjusted final BW, ADG, and G:F were not impacted by dietary treatment (*P* ≥ 0.29). This is similar to what has been reported by Smock et al. (2020a) who noted no improvements in cumulative growth performance responses in steers when *B. subtilis* PB6 was fed to high-stressed feeder steers. Alternatively, Smock et al. (2020a) noted an improvement in ADG and DMI during the initial 56-d feedlot-receiving phase when *B. subtilis* PB6 was supplemented to high-stressed feeder steers. Estimated dietary NE based on growth performance was in close agreement with the expectation based on diet formulation (*P* ≥ 0.46). It has been reported previously that *B. subtilis* supplementation increased the ADG of broiler chicks (Sen et al., 2012). Others have reported that *B. subtilis* PB6 supplementation increased DMI when weaned Holstein steers were experimentally infected with *Salmonella* (Broadway et al., 2020). Improvements in feed conversion efficiency have been reported by others in broiler chicks and feedlot steers when *B. subtilis* was fed compared to nonsupplemented controls (Sen et al., 2012; Zhang et al., 2013; Kemin, 2018). No appreciable differences for animal growth performance responses in the present study is likely due to the steers being under minimal amounts of environmental stress during the course of the study.

**Table 3.** Cumulative growth performance responses

	Treatment^*a*^			
Item	CON	CLO	SEM	*P*-value
Pens, *N*	12	12	–	–
Steers, *N*	119	119	–	–
Days on feed	140	140	–	–
Initial BW^*b*^, kg	402	401	1.29	0.37
Live basis				
Final BW^*b*^, kg	646	638	4.4	0.06
ADG, kg	1.75	1.69	0.029	0.07
DMI, kg	11.41	11.36	0.098	0.63
ADG/DMI (G:F)	0.153	0.149	0.0025	0.11
Carcass-adjusted basis				
Final BW^*c*^, kg	657	653	4.2	0.29
ADG, kg	1.82	1.80	0.027	0.39
DMI, kg	11.41	11.36	0.098	0.63
G:F	0.160	0.158	0.0026	0.58
Observed dietary NE, Mcal/kg				
Maintenance	2.04	2.03	0.020	0.46
Gain	1.38	1.37	0.018	0.46
Observed/expected dietary NE^*d*^				
Maintenance	0.99	0.98	0.011	0.49
Gain	1.00	0.99	0.013	0.46

^*a*^Fed no probiotic (CON) or fed 0.5 g/steer/d of *B. subtilis* PB6 (CLOSTAT 500, Kemin Industries, Des Moines, IA; CLO).

^*b*^A 4% pencil shrink was applied to account for gastrointestinal tract fill.

^*c*^Calculated as HCW/0.625.

^*d*^Observed dietary NE/tabular trial NE, where tabular trial NEm was 2.05 Mcal/kg and NEg was 1.38 Mcal/kg.

### Carcass Characteristics

Carcass trait responses are located in Table 4. Previous data with regards to *B. subtilis* supplementation to feedlot finishing cattle is limited. There were no differences (*P* ≥ 0.15) among treatments for any carcass traits measured in the present experiment. Moreira et al. (2016) indicated that Nellore bulls supplemented with 10/bull/d of calcium butyrate (ButiPEARL, Kemin Industries) and 10 g/bull/d of *B. subtilis* (CLOSTAT, Kemin Industries) had greater intramuscular fat accumulation compared to cattle not supplemented with *B. subtilis* but indicated no differences in any other carcass parameters. In transit-stressed steers from the southeastern United States transported and fed in Oklahoma, the supplementation of *B. subtilis* PB6 (CLOSTAT, Kemin Industries) had no influence on HCW, dressing percentage, RF, REA, USDA marbling score, or calculated YG (Kemin, 2018). Smock et al. (2020a) detected no differences for HCW, dressing percentage, USDA marbling scores, RF, REA, or calculated YG when *B. subtilis* PB6 was fed to finishing steers. The distribution of USDA low choice grade tended (*P* = 0.08) to be greater for CLO compared to CON steers. There were no other differences (*P* ≥ 0.12) among treatments for the distribution of USDA yYG or quality grade in the present study. Finally, there were no treatment effects (*P* ≥ 0.54) for the prevalence of abscessed livers in this experiment. These findings are very similar to Smock et al. (2020a), who indicated that the distribution of USDA YG and quality grade or condemned livers were not influenced by the supplementation of *B. subtilis* PB6 to finishing beef steers.

**Table 4.** Carcass trait responses

	Treatment^*a*^			
Item	CON	CLO	SEM	*P*-value
Pens, *N*	12	12	–	–
Steers, *N*	119	119	–	–
HCW, kg	411	408	2.6	0.29
Dressing percentage^*b*^, %	63.56	64.01	0.333	0.19
Rib fat, cm	1.37	1.32	0.034	0.15
Ribeye area, cm2	87.10	86.77	1.078	0.79
Marbling^*c*^	442	438	13.8	0.77
KPH, %	1.71	1.71	0.019	0.97
Calculated YG^*d*^	3.31	3.25	0.067	0.38
Retail yield^*e*^, %	49.92	50.04	0.145	0.41
Estimated EBF^*f*^, %	30.71	30.39	0.245	0.22
Final BW at 28% EBF^*f*^, kg	600	601	5.4	0.88
USDA YG distribution				
YG 1, %	0.0	0.0	–	–
YG 2, %	27.4	23.0	4.03	0.46
YG 3, %	53.8	64.4	4.46	0.12
YG 4, %	18.8	12.6	3.39	0.22
YG 5, %	0.0	0.0	–	–
USDA quality grade distribution				
Select, %	41.9	36.0	5.51	0.43
Low choice, %	32.3	44.9	4.68	0.08
Average choice, %	17.0	15.5	3.77	0.75
High choice, %	7.1	3.6	2.95	0.42
Prime, %	1.7	0.0	0.79	0.17
Liver abscess scores^*g*^				
Normal, %	67.4	65.1	5.62	0.77
A−, %	12.9	15.3	4.11	0.68
A, %	9.4	6.9	2.48	0.48
A^+^, %	10.3	12.7	2.96	0.54

^*a*^Fed no probiotic (CON) or fed 0.5 g/steer/d of *B. subtilis* PB6 (CLOSTAT 500, Kemin Industries, Des Moines, IA; CLO).

^*b*^Calculated as HCW/(final BW pencil shrunk 4%).

^*c*^400 = small^00^ (USDA low choice).

^*d*^Calculated according to the USDA regression equation (USDA, 1997).

^*e*^As a percentage of HCW according to Murphey et al. (1960).

^*f*^Calculated according the equations described by Guiroy et al. (2002).

^*g*^According to the Elanco Liver Scoring System: normal (no abscesses), A− (one or two small abscesses or abscess scars), A (two to four well-organized abscesses of less than 2.54 cm diameter), or A+ (one or more large active abscesses of greater than 2.54 cm diameter with inflammation of surrounding tissue).

### Salmonella Prevalence

*Salmonella* prevalence data are located in Table 5. There was no *Salmonella* recovered from any fecal samples collected on study days 1, 28, or 56 (6, 34, or 62 d following arrival to the SERF). On study day 112 (118 d following arrival to the SERF), there was numerically greater (*P* = 0.17; 25.0 vs. 8.3%) fecal prevalence of *Salmonella* in CON steers compared to CLO steers. Study day 112 was during a heat event that occurred in the Northern Plains and Midwest region (Table 2); this could potentially explain why *Salmonella* was detected in the feces on day 112. On day 140 of the present study, there was numerically greater (*P* = 0.34; 0.0 vs. 8.3%) fecal *Salmonella* prevalence for CLO compared to CON steers. Smock et al. (2020b) noted an appreciable decrease in fecal *Salmonella* prevalence in high-stressed feeder steers supplemented with *B. subtilis* PB6 on day 28 of the feedlot-receiving period. However, no differences among treatments for fecal *Salmonella* incidence were noted on day 196 of the feeding period (Smock et al., 2020b). The lack of detectable *Salmonella* in these steers could be due to the fact that the steers used in the present experiment were not transitioned through a cattle auction facility. Thus, the steers used in the present experiment experienced minimal marketing stress and minimal environmental stressors during the present study, which could have reduced *Salmonella* exposure and/or shedding (Gragg et al., 2013). Additionally, steers from the Northern Plains region of the United States (e.g., South Dakota) have been shown to have no *Salmonella-*positive lymph nodes in finished cattle upon harvest (Gragg et al., 2013), suggesting regional differences in *Salmonella* prevalence in fed-cattle populations (Green et al., 2010; Gragg et al., 2013). Regional differences in fecal and subiliac lymph node *Salmonella* prevalence in beef cattle should be investigated further. Regional differences in *Salmonella* prevalence should be exploited by cattle feeders and this might provide opportunities to increase cattle feeding numbers in specific regions of the United States, such as the Northern Great Plains.

**Table 5.** *Salmonella* prevalence in finishing steers

	Treatment^*a*^			
Item	CON	CLO	SEM	*P*-value
Pens, *N*	12	12	–	–
Steers, *N*	60	60	–	–
Fecal *Salmonella* prevalence,^*b*^ %				
Day 1 (6 d)	ND	ND	–	–
Day 28 (34 d)	ND	ND	–	–
Day 56 (62 d)	ND	ND	–	–
Day 112 (118 d)	25.0	8.3	10.95	0.17
Day 140 (146 d)	0.0	8.3	5.89	0.34
Subiliac lymph node *Salmonella* prevalence,^*b*^ %			–	–
Days of harvest (147 d)	ND	ND	–	–

ND, none detected.

^*a*^Fed no probiotic (CON) or fed 0.5 g/steer/d of *B. subtilis* PB6 (CLOSTAT 500, Kemin Industries, Des Moines, IA; CLO).

^*b*^Days of study (days following arrival to the feedlot).

## CONCLUSION

These data indicate that *B. subtilis* PB6 had no influence on feedlot phase growth performance, efficiency of dietary NE utilization, or carcass traits. If *Salmonella* is determined to be an adulterant in raw beef products, then cattle feeders might be able to exploit regional differences in *Salmonella* prevalence and regional-based assessment of identified feed additives that have proven efficacy to mitigate *Salmonella* prevalence in beef cattle should be conducted.

*Conflict of interest statement*. No potential conflict of interest is reported by Z.K.S., P.R.B., K.R.U., W.C.R., J.A.W., N.C.B.S., or J.A.C. other than the fact that Kemin Industries provided funding for this research; D.L. is a consultant for Kemin Industries and J.E.H. is employed by Kemin Industries.

## Notes

Kemin Industries provided funding for this research.
